# Enhanced Immune Responses and Protective Immunity to Zika Virus Induced by a DNA Vaccine Encoding a Chimeric NS1 Fused With Type 1 Herpes Virus gD Protein

**DOI:** 10.3389/fmedt.2020.604160

**Published:** 2020-12-03

**Authors:** Lennon Ramos Pereira, Rúbens Prince dos Santos Alves, Natiely Silva Sales, Robert Andreata-Santos, Aléxia Adrianne Venceslau-Carvalho, Samuel Santos Pereira, Maria Fernanda Castro-Amarante, Mônica Josiane Rodrigues-Jesus, Marianna Teixeira de Pinho Favaro, Rosa Maria Chura-Chambi, Ligia Morganti, Luís Carlos de Souza Ferreira

**Affiliations:** ^1^Laboratory of Vaccine Development, Department of Microbiology, Institute of Biomedical Sciences, University of São Paulo, São Paulo, Brazil; ^2^Biotechnology Center, Institute of Energy and Nuclear Research (IPEN), São Paulo, Brazil

**Keywords:** Zika virus, NS1 protein, gD protein, DNA vaccine, HSV-1, flavivirus

## Abstract

Zika virus (ZIKV) is a globally-distributed flavivirus transmitted to humans by *Aedes* mosquitoes, usually causing mild symptoms that may evolve to severe conditions, including neurological alterations, such as neonatal microcephaly and Guillain-Barré syndrome. Due to the absence of specific and effective preventive methods, we designed a new subunit vaccine based on a DNA vector (pgDNS1-ZIKV) encoding the non-structural protein 1 (NS1) genetically fused to the Herpes Simplex Virus (HSV) glycoprotein D (gD) protein. Recombinant plasmids were replicated in *Escherichia coli* and the expression of the target protein was confirmed in transfected HEK293 cells. C57BL/6 and AB6 (IFNAR1–/–) mice were i.m. immunized by electroporation in order to evaluate pgDNS1-ZIKV immunogenicity. After two doses, high NS1-specific IgG antibody titers were measured in serum samples collected from pgDNS1-ZIKV-immunized mice. The NS1-specific antibodies were capable to bind the native protein expressed in infected mammalian cells. Immunization with pgDNS1-ZIKV increased both humoral and cellular immune responses regarding mice immunized with a ZIKV NS1 encoding vaccine. Immunization with pgDNS1-ZIKV reduced viremia and morbidity scores leading to enhanced survival of immunodeficient AB6 mice challenged with a lethal virus load. These results give support to the use of ZIKV NS1 as a target antigen and further demonstrate the relevant adjuvant effects of HSV-1 gD.

## Introduction

Zika virus (ZIKV) is an arthropod-borne virus with a positive single-stranded RNA that codes for three structural and seven non-structural proteins (1). While the ZIKV classical transmission cycle mainly involves *Aedes* mosquitos (1, 2), intrauterine and sexual transmission routes have also been demonstrated (3). According to the WHO, ZIKV has been reported in more than 80 countries. Infections related to this virus causes a plethora of symptoms ranging from flu-like symptoms such as fever, rash, conjunctivitis, headache and eye pain to severe forms such as Guillan-Barré Syndrome (GBS) and Zika Congenital Syndrome (CZS) (4). The latter is a set of malformations that deeply impact the development of newborns and may lead to microcephaly (5–7). Despite the burden of the disease and the urge to develop approaches to prevent virus dissemination, there are no licensed therapies or vaccines to ZIKV infection so far.

Despite the absence of a clear protection correlate against ZIKV infection, recent evidences indicate that an optimal ZIKV vaccine should induce both cellular and humoral immune responses (8–10). The induction of ZIKV-specific neutralizing antibodies (nAb) by vaccines was capable to confer protection in mice and non-human primates (NHP). In addition, passive immunization with sera isolated from vaccinated or infected NHP or humans conferred protection under experimental conditions (11–15). On the other hand, T cell mediated immune responses also contribute to the control of ZIKV infection. Increased viral loads and mortality rates are observed in CD8^+^ T cell-deficient mice while transfer of ZIKV-specific CD8^+^T cells to T cell deficient mice reduced infection and conferred protection (16–18). Recently, the role of type I CD4^+^ T helper cells has also shown to contribute to the control of ZIKV infection (19–21). Further published evidences repeatedly demonstrated that both nAbs and T cells responses contribute to protective immunity to ZIKV infection (14, 19, 22–24).

The abnormally high incidence of GBS and CZS in certain locations raised the question whether the pre-existing immunity to other flavivirus, such as DENV, could be implicated in such a phenomenon, likely through Antibody-Dependent Enhancement (ADE). It is well-known that ZIKV and DENV share antigen cross-reactivity at both antibody (Ab) and T cell levels, and since Abs can play a dual role in protection and in DENV pathogenesis, the same might be true for ZIKV (17, 25). In fact, studies using mouse models and, more recently, human data have already shown a direct role of anti-structural protein Abs in both DENV and ZIKV pathogenesis (26–28). Despite these evidences, most ZIKV vaccines tested under experimental conditions target generation of neutralizing antibodies against structural antigens (13, 14, 29–33). An alternative would be the use of non-structural proteins in vaccine formulations, since these antigens may trigger both B and T cell protective responses without the undesirable risks of ADE (9).

The flavivirus non-structural proteins 1 (NS1) are glycoproteins, with molecular weight ranging from 46 to 55 kDa, implicated in several mechanisms, such as replication, negative RNA strand synthesis and evasion from the host's immune response (34). NS1 is highly immunogenic and may be associated to the cytoplasmic membrane as dimers or hexamers that are secreted to extracellular medium. It has been reported that anti-NS1 Abs target infected cells and induce virus clearance by Antibody-dependent Cytotoxicity (ADCC) and deposition of complement system proteins. In addition, ZIKV-infected cells may also be targeted by T cells through MHC presentation of NS1-derived epitopes. In fact, both immunological responses generate protective immunity to ZIKV. Anti-ZIKV vaccine strategies based on recombinant vesicular stomatitis virus (rVSV) (35), DNA vaccines (23, 36) or Modified Vaccinia Ankara virus (37) showed different protection levels by inducing either one or both humoral and cellular responses. Furthermore, mice and human isolated NS1-specific monoclonal antibodies (mAbs) showed Fc-dependent protection to ZIKV challenges, including non-pregnant and pregnant mice (38, 39). These results show that ZIKV NS1 protein is a suitable antigen for vaccines based on different delivery platforms.

Previous studies showed that the genetic fusion of antigens at the C-terminal region of the herpes simplex virus type I (HSV-1) glycoprotein D (gD) may improve the induction of antigen-specific humoral and cellular immune responses, either based on the DNA vaccine platform or as purified recombinant protein-based vaccines (40–42). The HSV-1 gD is capable to bind with high affinity to several antigen presenting cell receptors, including nectin-1, and the herpes virus entry mediator (HVEM) (43), which is a member of the tumor necrosis factor receptor (TNFR) family. Interaction of gD and HVEM induces NF-κB RelA expression in a TRAF2-dependent-way, resulting in pro-survival signals within activated T cells (44, 45). The binding of gD to HVEM induces strong immunomodulatory effects on T cells as previously demonstrated by our group and others (40, 41, 46–49).

In this study we designed and tested a DNA vaccine based on expression of a chimeric ZIKV NS1 protein genetically fused to the HSV-1 gD protein in order to enhance the anti-NS1 immune responses and protective immunity to ZIKV. Immunization of immunocompetent mice with the DNA-vaccine induced increased serum NS1-specific IgG titers as well as NS1-specific IFNγ-secreting cells, in comparison with mice immunized with a vector encoding only NS1. The presence of gD was also correlated to the reduced morbidity and mortality induced in immunodeficient AB6 mice challenged with ZIKV. These results support the use of the NS1 antigen in anti-ZIKV vaccine strategies and validate the adjuvant effects associated with co-expression of HSV-1 gD protein.

## Materials and Methods

### Ethics Statement

Male or female C57BL/6 (6–8 weeks-old) or AB6 (IFNAR1–/–) (4 weeks-old) mice were bred under specific pathogen-free conditions at the Isogenic Mouse Facility of the Microbiology Department, University of São Paulo, Brazil. The protocols were approved by the Institutional Animal Care and Use Committee (CEUA) of the University of São Paulo (protocol number 96/2016) and conducted according to the Ethical Principles of Animal Experimentation established by the Brazilian College of Animal Experimentation. Animal group size was determined to achieve 80% resolution power using the Experimental Design Assistant (EDA) platform (https://eda.nc3rs.org.uk/).

### Plasmids and Recombinant Proteins

**Figure 1:**
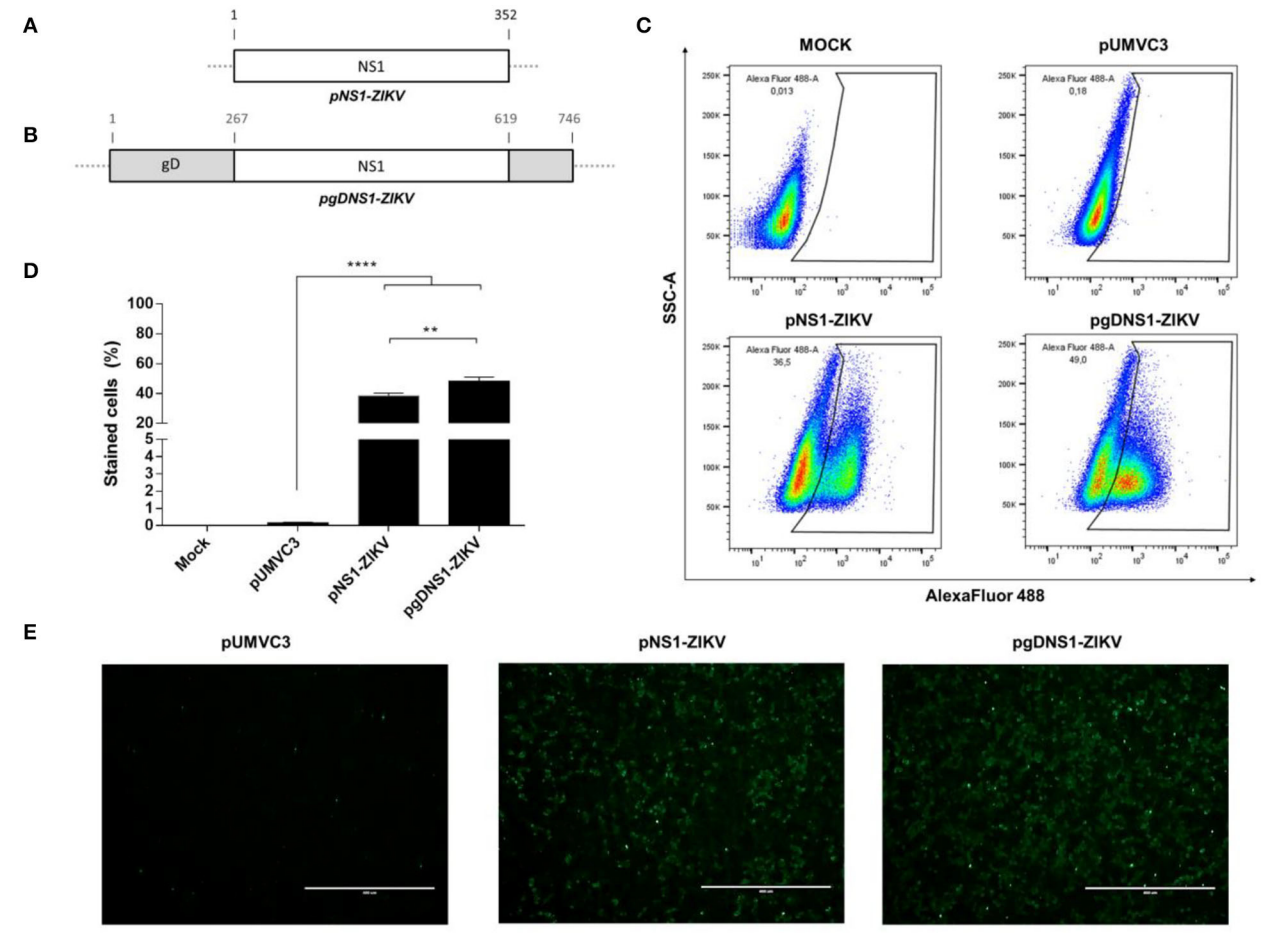
Expression of the recombinant ZIKV NS1 and the chimeric ZIKV NS1 and HSV-1 gD. **(A,B)** Schematic representations of pNS1-ZIKV, encoding the full length ZIKV NS1 protein **(A)** and pgDNS1-ZIKV, encoding the genetic fusion of NS1 and gD **(B)**. **(C–E)** Detection of the ZIKV NS1 expressed in HEK293-T cells 24 h after transfection with the tested plasmids. Cells were labeled with anti-ZIKV NS1 mAb followed by anti-mouse IgG antibodies conjugated to Alexa Fluor 488 and analyzed by flow cytometry **(C,D)** and immunofluorescence **(E)**. Data are expressed as mean ± SEM of two independent experiments performed in duplicate. Scale bar = 400 μm. Statistical significance was calculated using One-way ANOVA with post-test Bonferroni (**p < 0.01, ****p < 0.0001, NS = not significant).

The DNA vaccines encoding the full-length ZIKV NS1 (GenBank: ALU33341) isolated (pNS1-ZIKV) or as a chimeric protein genetically fused the HSV-1 gD protein (pgDNS1-ZIKV) were synthetized at GenScript, USA (Figure 1A). The synthetic genes were optimized to the human codon usage and designed to contain the restriction sites *PstI* and *BglII* at the N‘ and C‘ terminus, respectively. The sequences were cloned into a pUMVC3 vector (Aldevron, ND, USA), as previously described (47). For the pgDNS1-ZIKV construction the sequence of full-length ZIKV NS1 was flanked by *ApaI* restriction sites (Supplementary Figure 1). The *Escherichia coli* DH5α strain was transformed with the recombinant plasmids separately and the plasmid DNA was purified using the EndoFree Plasmid Mega kit (Cat.: 12183, QIAGEN), according to the manufacturer's instructions. The constructions were confirmed by restriction analyses and gene sequencing.

The ZIKV NS1 protein was expressed on the recombinant *E. coli* BL21-CodonPlus (DE3)-RIL cells and purified by affinity chromatography after *in vitro* refolding of the protein under high pressure conditions, as previously described (50).

### Virus and Cells Lines

The Brazilian clinical ZIKV isolate (ZIKV^BR^) (GenBank: KU729217.2) was obtained from Evandro Chagas Institute in Belém (Pará, Brazil). ZIKV^BR^ propagation was carried out in *Aedes albopictus* clone C6/36 cells cultured in Leibovitz L-15 medium (Vitrocell, Brazil) supplemented with 2% fetal bovine serum (FBS) (Life Technologies, USA), followed by a concentration protocol as previously described (5, 51). Vero CCL-81 cells were cultured in Minimum Essential Medium Eagle (MEM, Vitrocell) with 10% FBS. Human embryonic kidney cells (HEK-293 cells, ATCC No. CRL-11268) were cultured in Dulbecco's modified Eagle's medium (DMEM, Life Technologies) supplemented with 10% FBS.

### Detection of ZIKV NS1

Expression of ZIKV NS1 protein was detected either through immunofluorescence or flow cytometry. In both cases, HEK-293 cells were equally transfected with the plasmids according to a previously described protocol (52). Briefly, HEK-293 cells were seeded (10^5^ cell/well) in 24-wells plates (Corning, USA) and cultured at 37°C (5% CO_2_) until reaching 70–80% confluence. One hour before the transfection, the cell culture supernatant was removed and 200 μl/well of DMEM (supplemented with 2% FBS) was added to the cells. For transfection, MIX solutions containing 1 μg of the plasmids to 3 μg of polyethyleneimine (PEI) (Cat.: 408727, Sigma Aldrich, USA) were prepared in 150 mM NaCl solution. After incubation for 5 min at room temperature (RT), the mixtures were distributed evenly over the plates and the cells were incubated for up to 24 h (37°C, 5% CO_2_).

For detection of ZIKV NS1, transfected cell monolayers were washed (2×) with PBS and fixed with 4% formaldehyde diluted in PBS for 15 min (RT). After incubation, cells were permeabilized with 0.1% Triton X-100 in PBS (15 min at RT) and blocked with a blocking solution (BS) (2% BSA diluted in PBS) for 30 min at RT. After a new washing cycle (3×), the cells were stained with anti-ZIKV NS1 4H2 mAb (20 μg/ml) diluted in BS and kept for 1 h at room temperature (RT) under agitation. After washing (3×) with 0.05% Tween 20 in PBS (PBS-T), cells were incubated (45 min, at RT) with anti-mouse IgG antibody conjugated to AlexaFluor 488 (Cat.: A11001, Invitrogen, USA). Finally, cells were washed with PBS-T and observed under an immunofluorescence microscope (Evos FL Thermo Scientific, USA) with images captured at 100x magnification.

For flow cytometry assays, cell monolayers were washed 2× with PBS, trypsinized, and fixed/permeabilized with a Cytofix/Cytoperm kit (BD Bioscience, USA), according to the manufacturer's instructions. Cells were, then, stained with the primary antibody 4H2 mAb (5 μg/ml) on ice for 30 min. After washing (2×), cells were incubated (30 min on ice) with the anti-mouse conjugated to Alexa Fluor 488 (Cat.: A11001, Thermo Fisher Scientific) diluted 1:800. Finally, after washings (2×), the cells were suspended in 200 μl of PBS-2% FBS solution and analyzed by an LSR FortessaTM analyzer (BD, Franklin Lakes, NJ, USA). The data obtained were analyzed using FlowJo software (version 10, Tree Star, San Carlo, CA) to determine the percentage of stained cells.

### Mice and Immunization Regimen

C57BL/6 (5–7 animals/group) or AB6 (4–6 animals/group) mice were immunized with 2 doses of 50 μg of pUMVC3, pNS1-ZIKV, or pgDNS1-ZIKV formulated in apyrogenic saline (0.9%). For immunization, the animals were previously anesthetized with a mixture of Ketamine and Xylazine (100 and 10 mg/kg, respectively) administered intraperitoneally (i.p.). Then, the animals received intramuscular (i.m.) injections with the formulations followed by electroporation (two 130 V pulses with 1 ms duration and four 70 V pulses with 50 ms duration, with an interval of 450 ms between each pulse), which was carried out with the CUY560-5-0.5 electrode using the NEPA21 Super Electroporator (Nepa Gene Co, Japan.), at an interval of 2 weeks. Blood samples were obtained by submandibular plexus puncture 14 days after the administration of each dose and centrifuged at 3,000 g for 30 min to separate the sera. The obtained serum samples were stored at −20°C for future analysis.

### Enzyme-Linked Immunosorbent Assay (ELISA)

High-binding ELISA plates (Corning) were coated with purified ZIKV NS1 protein (200 ng/well) diluted in coating buffer (32.5 mM NaHCO_3_, 14.9 mM Na_2_CO_3_, pH 9.6) for 18 h at 4°C. The plates were washed three times with PBS containing 0.05% Tween-20 (PBS-T) and added with 200 μl/well of a blocking buffer (BB) containing 5% non-fat milk solution with 1% bovine serum albumin (BSA) in PBS-T. After incubation (2 h at 37°C), plates were washed (3×) and 100 μl of the individual serum samples, previously diluted (starting 1:25) in BB, were added to the wells and incubated for 1 h at 37°C. After a new wash cycle (3×), anti-mouse IgG antibody conjugated to horseradish peroxidase (HRP) (Sigma Aldrich) was added to the wells at 1:4,000 dilution in BB. For the determination of IgG subclasses, anti-mouse IgG1 or IgG2c HRP-conjugated antibodies (Southern Biotech, USA) were used. After incubation (1 h, 37°C), plates were washed (3×) and 100 μl of developing solution containing o-phenylenediamine dihydrochloride (OPD) (Sigma Aldrich) and H_2_O_2_ were added to each well and incubated for 15 min at RT in the dark. The reaction was stopped with 50 μl/well of 1 M sulfuric acid and the absorbance was measured at 492 nm (Ab_492nm_) with a plate reader (BioTek, Winooski, VT, USA). Antibody titers were determined as the reciprocal end dilution value of the serum sample at which the absorbance obtained was at least 0.1 units above of the cut-off value (mean + 2 SD of Ab_492nm_ obtained on wells containing no serum).

### Antibody Binding to Native NS1 Protein

Vero cells were seeded (5 × 10^4^ cell/well) in flat 96-well plates (Corning) and incubated for 24 h at 37°C (5% CO_2_). Established cell monolayers were infected with ZIKV^BR^ at multiplicity of infection (MOI) 1.0 for 24 h (37°C, 5% CO_2_). After infection, the monolayers were washed with PBS (2×), trypsinized (Vitrocell) and fixed/permeabilized with a Cytofix/Cytoperm kit (BD Bioscience) according to the manufacturer's instructions. The cells were labeled with pooled serum samples from immunized mice diluted 1:1,000 or 4H2 mAb (5 μg/ml) for 30 min on ice. After washing (2×), cells were stained for 30 min (on ice) with a goat anti-mouse IgG antibody conjugated to Alexa Fluor 488, diluted 1:800 (Cat.: A11001, Thermo Fisher Scientific). After new wash cycles (2×), the cells were suspended and analyzed in the LSR FortessaTM analyzer (BD, Franklin Lakes, NJ, USA). The data were analyzed using FlowJo software (version 10, Tree Star, San Carlo, CA) to determine the percentage of stained Vero cells.

### Peptide Synthesis

The peptides used in this work were synthesized at GenScript USA Inc. The prediction of immunogenic regions was carried out with algorithm (53, 54) hosted at the Immune Epitope Database (IEDB) Tools (http://tools.iedb.org/mhci/), using the amino acid sequence corresponding to the C-terminal portion of the native ZIKV NS1 protein (GenBank–ALU33341). Binding affinities were obtained for all 8–14 mer peptides for the H2-Kb and H2-Db alleles. The selected alleles had the classification of the consensus percentage restricted to 1.7% with a score ≤1. Two high-score-predicted peptides were selected, custom-made by GenScript (Piscataway, NJ, USA), suspended in DMSO and stored at −80°C before use.

### Analysis of Cellular Immune Response

Measurement of induced cellular immune responses was carried out according to a previously described adapted protocol (16). Briefly, 2 weeks after the last vaccine dose, the immunized mice were intravenously (i.v.) infected with 10^6^ plaque-forming units (PFU) of ZIKV^BR^. Three days after infection (d.p.i) the animals were euthanized, the spleens were harvested surgically under aseptic conditions and the splenocytes were isolated, as previously described (52). Then, the isolated spleen cells were stimulated *in vitro* with peptides derived from ZIKV NS1 and the number of IFNγ-producing cells was determined by enzyme-linked immune absorbent spot (ELISpot).

ELISpot was carried out according to a previously described protocol (55). Briefly, splenocytes from immunized and infected mice were seeded (2 × 10^5^ cells/well) in 96-well plates (Millipore, USA), previously coated with capture antibody to IFNγ (BD Biosciences, USA). Then, the cells were cultured for 48 h (37°C, 5% CO_2_) in the presence or absence of NS1-derived peptides (100 ng of each peptide/well). After stimulation, the plates were washed (3 × with PBS and 5 × with PBS-T) and stained (2 h at RT) with a biotinylated mouse anti-IFNγ mAb (BD Biosciences, USA) at 2 μg/ml. After washing, the plates were incubated with peroxidase-labeled streptavidin (Sigma, USA) for 2 h at RT. Plates were washed again and developed with TrueBlue™ Substrate (KPL, Milford, MA, USA) for 20 min at RT. The spots were counted and expressed as IFNγ-producing cells/10^6^ splenocytes.

### Lethal Challenge With the ZIKV^BR^

Two weeks after administration of the second vaccine dose, immunized AB6 mice were i.v. infected with 10^6^ PFU of ZIKV^BR^. The animals were monitored for up to 15 days for the appearance of clinical signs according to an arbitrary score scale (Healthy, score 0; ruffled fur, score 1; paralysis, score 2; deformed spinal column, score 3; moribund, score 4), measurement of body weight and mortality. Serum samples were collected on alternate days and stored at −80°C for analysis of viremia.

### Virus Titration

The number of infectious ZIKV particles was determined by plaque assay. Briefly, 10-fold serial dilutions of virus samples were prepared in MEM medium, which were added to the Vero cells monolayers previously established in 24-wells plates (Corning) and incubated for 1 h at 37°C. After incubation, the cell supernatants were removed by aspiration, an overlay solution containing MEM plus carboxymethylcellulose (1%) and SFB (2%) was added to the cells and incubated (37°C, 5% CO_2_) for 4 days. The cells monolayers were fixed with formaldehyde (4%) diluted in PBS for 15 min (RT). After washing with water, cells were stained with 1% violet crystal solution (Laborclin) for 10 min (RT). Viral lysis plates were counted and expressed as plaque form units per mL (PFU/ml).

### Statistical Analysis

Statistical analyses were performed using the Prism 6 software (GraphPad Software Inc, LA Jolla, CA). *T*-test was used to compare only two groups. One-way ANOVA was applied with Bonferroni's *post-hoc* test to compare results involving several (≥3) groups. Two-way ANOVA followed by Bonferroni's correction was used when the data involved several groups and more than one variable (time points). Log-rank test (Mantel-Cox) was used to analyze the survival and morbidity data. Differences were considered significant when the *p*-value (p) was ≤0.05.

## Results

### The DNA Vectors Encoding the ZIKV NS1 Protein

Two plasmids encoding the full-length ZIKV NS1 protein, either isolated (pNS1-ZIKV) (Figure 1A) or genetically fused to HSV-1 gD protein (pgDNS1-ZIKV) (Figure 1B), were constructed. Plasmids were submitted to restriction analyses with *BglII* and *PstI* and the released fragments had the expected electrophoretic mobilities (NS1–1,056 pb; gDNS1–2,262 pb) (Supplementary Figure 1). The expression of the encoded proteins in HEK 293 cells transfected with pNS1-ZIKV or pgDNS1-ZIKV was confirmed with NS1-specific antibodies by flow cytometry and immunofluorescence (Figures 1C,E). Interestingly, cells transfected with pgDNS1-ZIKV showed enhanced expression of ZIKV NS1 with regard to cells transfected with pNS1-ZIKV (Figure 1D). The expression of the HSV-1 gD in pgDNS1-ZIKV-transfected cells was also confirmed by immune blots (Supplementary Figure 2). Taken together, these results demonstrate that the recombinant proteins encoded by the DNA vaccines were properly expressed in mammalian cells.

### Fusion of ZIKV NS1 to HSV-1 gD Enhances the Induced Humoral and Cellular Immune Responses

**Figure 2:**
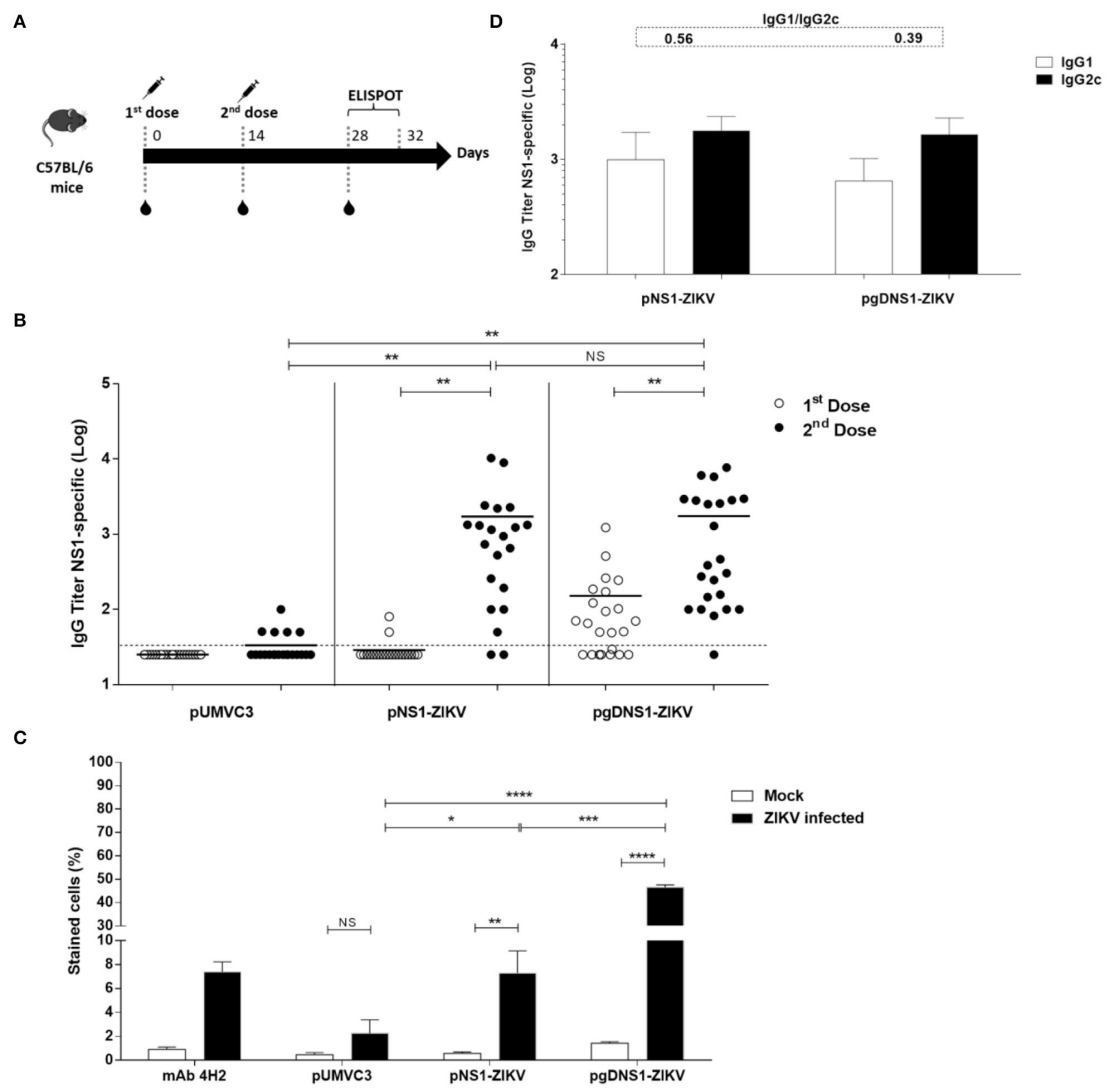
Immune responses elicited in vaccinated immunocompetent mice. **(A)** Schematic representation of the vaccination regimen. C57BL/6 mice were i.m. immunized with two doses (50 μg/animal) of DNA vaccines administered with electroporation. Serum samples were collected 14 days after each vaccine dose and the specific T cell responses were measured 2 weeks after the last vaccine dose by ELISpot. **(B)** Anti-NS1 IgG dose-response profiles measured by ELISA with individual serum samples collected from vaccinated mice (pUMVC3 and NS1-ZIKV, *n* = 21/each group; pgDNS1-ZIKV, *n* = 23). Data are expressed as means (bars) and individual (symbols) IgG titers (Log10) obtained from three independent experiments. **(C)** Binding of anti-NS1 serum antibodies to the native ZIKV NS1 protein. Diluted pooled serum samples collected from vaccinated mice (*n* = 10) were reacted with ZIKV-infected VERO cells and analyzed by flow cytometry. The 4H2 mAb (specific to NS1) was used as a control. **(D)** IgG subclass responses measured 14 days after the second vaccine dose in the individual serum samples from immunized mice (*n* = 10). The IgG1/IgG2c ratios are indicated at the top of the figure. Statistical significance was calculated using Two/One-way ANOVA with Bonferroni correction. *T*-test was used to analyze the IgG subclass responses (*p < 0.05, **p < 0.01, ***p < 0.001, ****p < 0.0001, NS = not significant).

Next, we accessed the immunogenicity of the DNA vaccines in wild-type (WT) C57BL/6 mice. The animals were immunized with two i.m. doses by electroporation (Figure 2A). As observed in Figure 2B, similar serum NS1-specific IgG titers were detected 14 days after the second vaccine dose in mice immunized with pNS1-ZIKV or pgDNS1-ZIKV. Analyses of the NS1-specific serum IgG subclass responses indicated that vaccinated mice elicited similar IgG1 and IgG2c subclass responses (Figure 2D). We also investigated the binding of pooled anti-NS1 serum samples to native NS1 expressed in the ZIKV-infected Vero cells. As shown in Figure 2C and Supplementary Figure 3, anti-NS1 antibodies raised in the mice immunized with pgDNS1-ZIKV showed higher cell binding activity with regard to serum samples collected from mice immunized with pNS1-ZIKV. As expected, mice immunized with the pUMVC3 vector did not elicit anti-NS1 antibody responses (Figures 2B,C).

**Figure 3:**
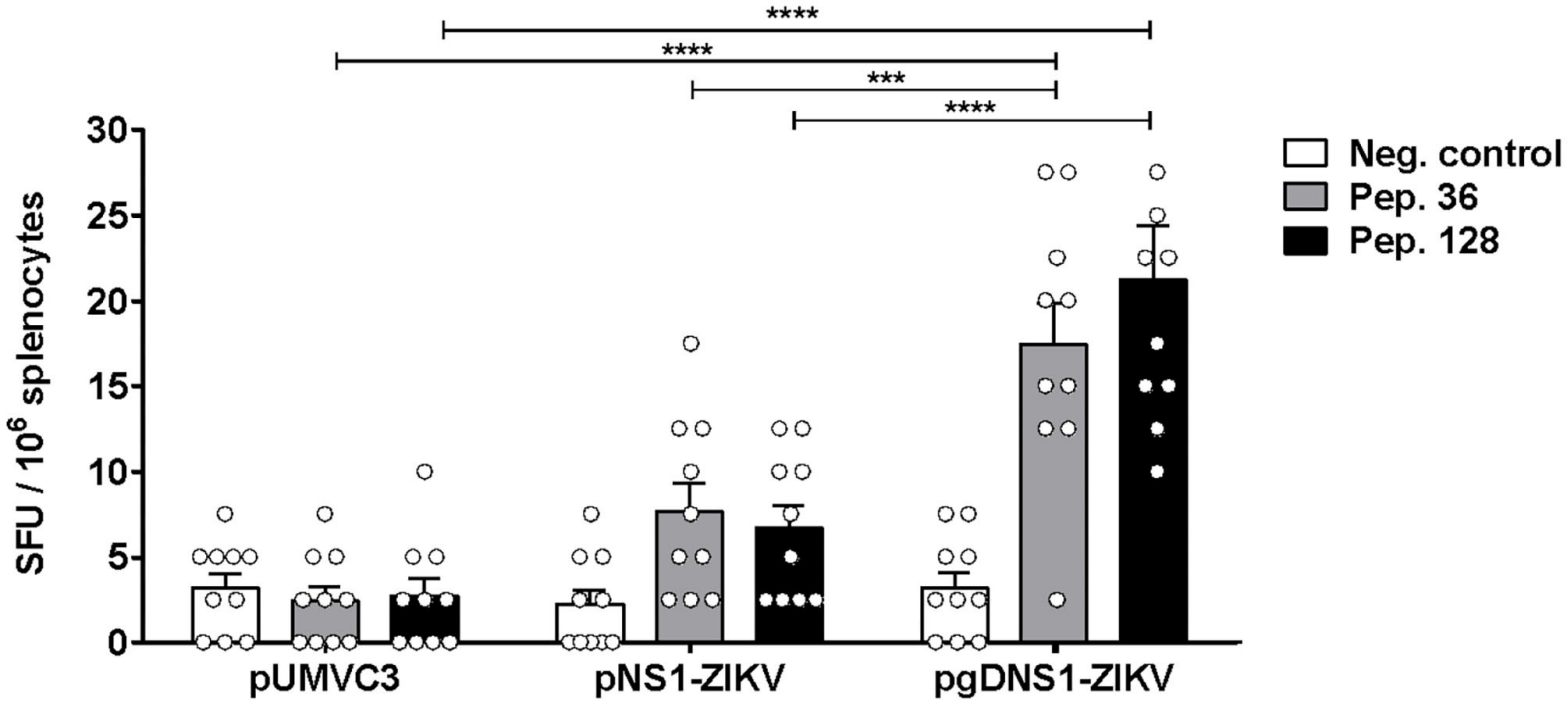
Analysis of cellular immune responses elicited in vaccinated immunocompetent mice. Immunized mice (*n* = 10) were infected with ZIKV^BR^ and the splenocytes were harvested 3 d.p.i. Isolated cells were stimulated *in vitro* with ZIKV NS1 peptides and analyzed regarding cytokine production via IFNγ-ELISpot. Data are presented as mean ± SEM (bars) or individual (symbols) amounts of spots. Statistical significance was calculated using Two-way ANOVA with Bonferroni's correction (***p < 0.001, ****p < 0.0001).

Fusion of antigens to HSV-1 gD has been shown to increase cellular immune responses to passenger antigens in vaccinated mice (40, 46–48). Thus, we also evaluated the antigen-specific cellular responses induced in mice immunized with the vaccine formulations. For that purpose, *in silico* predicted MHC-I restrict peptides of ZIKV NS1 were validated with splenocytes of ZIKV-infected mice (Supplementary Figure 4). The peptide prediction was performed using the C-terminal region of NS1 protein based on a recent report describing the presence of immunodominant CD8+ T cell epitopes in mice (23). Moreover, in order to maximize the detection of NS1-specific T cells, immunized mice were infected with ZIKV and, 3 days later, the numbers of IFNγ-producing cells were determined. This rationale was based on previous evidences demonstrating that expansion of antigen-specific CD8^+^ T cells may be accessed at this time-point after virus infection (16). Thus, the selected peptides (Supplementary Table 1) were applied in IFNγ-ELISPOT assays carried out with splenocytes harvested at 3 d.p.i from immunized mice (Figure 2A). As shown in Figure 3, only mice immunized with pgDNS1-ZIKV elicited statistically significant enhancement in the number of IFN-γ secreting spleen cells after *in vitro* stimulation with two different MHC-I restricted peptides. Taken together, these results indicate that immunization with pgDNS1-ZIKV simultaneously improves NS1-specific IgG response and T cell responses.

### ZIKV-Infected Immunodefient Mice Showed Reduced Viremia After Immunization With NS1-Encoding DNA Vaccines

**Figure 4:**
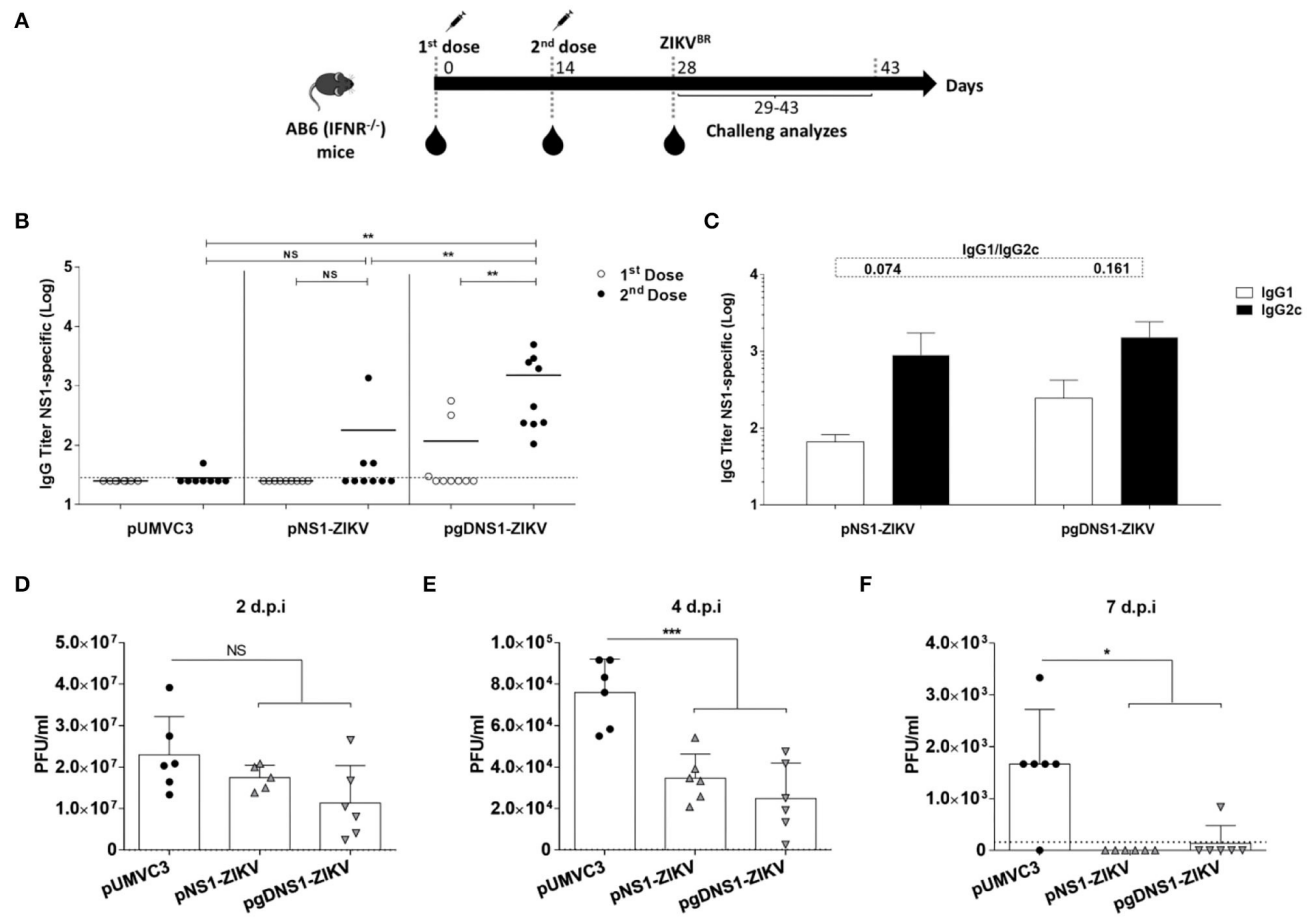
Immune responses and protective immunity induced by the DNA vaccines in immunodeficient mice. **(A)** Schematic representation of the vaccination regimen. AB6 mice were immunized twice by i.m. route in association with electroporation. Serum samples were collected 14 days after each vaccine dose. Mice were i.v. infected with 10^6^ PFU of ZIKV^BR^ 2 weeks after the last vaccine dose and monitored for viremia, morbidity and mortality for 15 days. **(B)** Total and **(C)** IgG subclass anti-NS1 responses were measured by ELISA with serum samples collected from immunized mice (*n* = 9). Data were obtained from three independent experiments and are expressed as mean (bars) ± SEM or individual values (symbols) of antibodies titers (Log10). The IgG1 and IgG2c ratios are indicated at the top of the figure. **(D–F)** 2 weeks after the last dose, immunized mice (*n* = 5) were i.v. challenged with ZIKV (10^6^ PFU/animal). Viremia is expressed in mean ± SD of PFU/ml, measured individually until day seven after infection. The results are representative of three independent experiments. Statistical significance was calculated using Two/One-way ANOVA with Bonferroni correction. *T*-test was used to analyze the IgG subclass results (*p < 0.05, **p < 0.01, ***p < 0.001, NS = not significant).

Since wild type mice are not susceptible to ZIKV, we tested the protective immunity induced by the NS1-encoding DNA vaccines in the AB6 mouse strain, deficient in the expression of type I IFN receptor (IFNAR1–/–) (56, 57). AB6 mice were immunized following the same regimen with 2 i.m. doses 2 weeks apart, and, 2 weeks after the last dose, animals were challenged with i.v. inoculation of 10^6^ PFU of ZIKV^BR^ (Figure 4A). Before the virus challenge, higher anti-NS1 serum IgG antibodies were detected in mice immunized with pgDNS1-ZIKV when compared to mice immunized with pNS1-ZIKV (Figure 4B). Notably, mice immunized with pNS1-ZIKV or pgDNS1-ZIKV showed prevailing IgG2c subclass responses (IgG1/IgG2c ratios of 0.074 and 0.161, respectively) (Figure 4C). Viremia was followed in vaccinated and challenged mice up to 7 days. As shown in the Figures 4D–F, mice immunized with the NS1-encoding DNA vaccines presented reduction of viremia with regard to mice immunized with pUMVC3 group. Similarly, mice immunized with either pNS1-ZIKV or pgDNS1-ZIKV showed a shorter viremia period (Figures 4D–F). These results clearly demonstrate that immunization with DNA vaccines encoding ZIKV NS1 has a direct impact on the length of the period and intensity of the viremic state, as measured in vaccinated AB6 mice.

### Immunization With pgDNS1-ZIKV Increased Anti-ZIKV Protective Immunity in AB6 Mice

**Figure 5:**
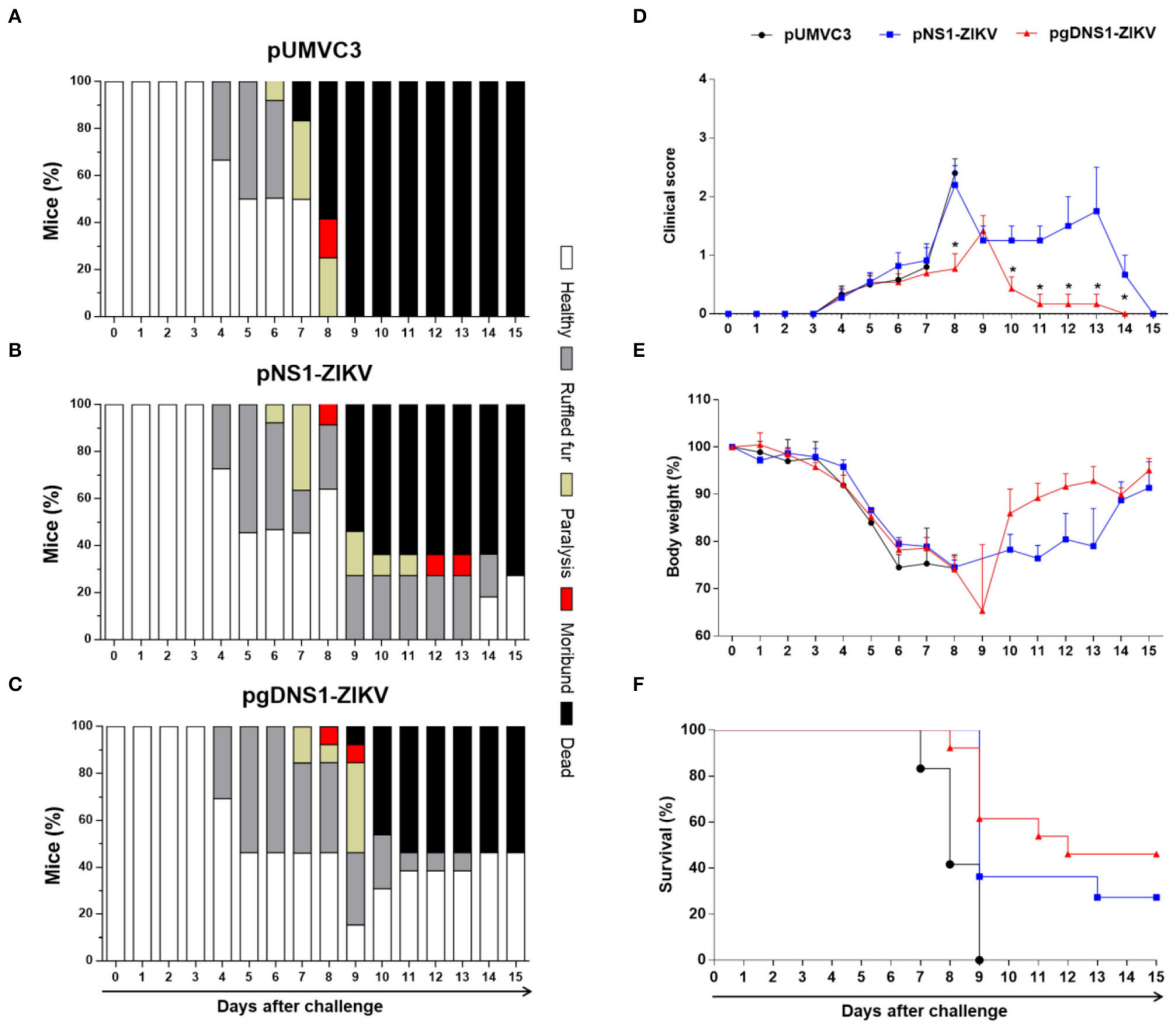
Protective immunity induced by ZIKV NS1-encoding DNA vaccines. AB6 mice were immunized with pUMVC3 (*n* = 12) **(A)**, pNS1-ZIKV (*n* = 11) **(B)** or pgDNS1-ZIKV (*n* = 13) **(C)** and monitored daily for up to 15 days after the ZIKV challenge. The results are represented as the percentage of animals classified with the clinical score condition (Healthy, ruffled fur, paralysis, moribund, or dead), in a color scale, as indicated in the figure. Clinical score **(D)**, body weight change **(E)** and mortality **(F)** profiles were evaluated in the challenged animals. The data represent results of three independent experiments and are presented as means ± SEM of percentage of the respective parameters followed in the different immunization groups. Statistical significance was evaluated using Two-way ANOVA with Bonferroni's correction. Mantel-Cox test was used to analyze the survival results. (*p < 0.001).

Immunized AB6 mice were monitored for vaccine-induced protection to ZIKV-induced morbidity and mortality. Using a symptom scale classification, mice immunized with pgDNS1-ZIKV showed reduced morbidity compared to non-vaccinated mice than animals immunized with pNS1-ZIKV (Figures 5A–D). pgDNS1-ZIKV vaccinated mice recovered body weight in ~10 days post infection, while those immunized with pNS1-ZIKV recovered their body weight 2 weeks after the challenge (Figure 5E). We also measured the vaccine induced anti-ZIKV protection in AB6 mice challenged with a lethal i.v. virus dose. As indicated in the Figure 5F, immunization with pgDNS1-ZIKV conferred higher survival (46%) to lethal ZIKV infection compared to mice immunized with pNS1-ZIKV (27%). No protection was observed in mice immunized with the control vector. Taken together, these results indicated that DNA vaccines encoding ZIKV NS1 confer partial protection to ZIKV infection in immunodeficient AB6 mice. In addition, the present results demonstrated that genetic fusion of NS1 to gD protein improves the induction of antigen-specific immune responses and the protective immunity to ZIKV.

## Discussion

Recent advances demonstrate that both cellular and humoral immune responses play important roles in the development of protective immunity to flavivirus under experimental and clinical conditions. Therefore, ideal vaccines for diseases associated with DENV and ZIKV infections shall preferentially activate both arms of the immune system (19, 58, 59). Here, we describe the use of a novel DNA vaccine strategy based on the expression of ZIKV NS1, either alone or genetically fused to the HSV-1 gD protein, as an approach to evaluate the protective role of anti-NS1 specific immune responses under experimental conditions. The results clearly demonstrated that immunization with NS1-encoding DNA vaccines induce NS1-specific immune responses in immunocompetent and immunodeficient mice leading to partial protection against lethal systemic ZIKV challenge in immunodeficient AB6 mice. The use of electroporation as a mean to deliver the DNA vaccines resulted in similar serum anti-NS1 IgG titers in mice immunized with pgDNS1-ZIKV or pNS1-ZIKV. Nonetheless, fusion of ZIKV NS1 to gD enhanced the binding of antibodies to the native NS1 antigen expressed in infected mammalian cells. Moreover, using splenocytes from pgDNS1-ZIKV-vaccinated mice we detected higher numbers of IFN-γ-secreting cells after *in vitro* stimulation with MHC-I-restricted NS1-derived peptides. More relevantly, immunization of immune deficient mice with the gD-NS1-encoding DNA vaccine reduced with more efficiency the morbidity and mortality rates detected after challenge with ZIKV. Collectively, these results support the use of genetic fusion of antigens to HSV-1 gD as promising platform for the development of ZIKV vaccine strategies and validate the adjuvant effects of the gD protein on the immune responses induced by ZIKV NS1-based DNA vaccines.

The potential of NS1 as a target antigen has been investigated regarding induction of both humoral and cellular immune responses (23, 35, 37, 60). This protein is targeted to circulating anti-NS1 antibodies either in plasma, as hexamers, or at cytoplasmic membrane of infected cells, as dimers, which may lead to cell lysis (38). Since NS1 protein is not present on the virus particle, anti-NS1 antibodies are not capable to induce the ADE phenomenon (36, 38, 39, 61). Although anti-DENV-NS1 antibodies have been implicated to side effects associated with cross-reactivity with host proteins (62–64), such effects seems to be virus specific, since no similar effects have been reported with Japanese encephalitis virus (JEV) anti-NS1 antibodies (64). Moreover, there are conflicting evidences regarding the protective and deleterious effects associated with NS1-specific antibodies both in DENV and ZIKV infections (65, 66). On the other hand, passive immunization of mice with polyclonal or monoclonal anti-NS1 antibodies promoted a clear protective effect to virus infection (36, 38, 39). Recently, a vaccinia ankara virus (MVA) modified to express the ZIKV NS1 induced protection after an intracranial challenge by inducing NS1-specific IgG2a and polyfunctional NS1-specific CD8^+^ T cells (35, 37). Human-derived anti-NS1 mAbs were also found to confer partial protection to immunodeficient ZIKV-challenged mice through passive immunity (38, 39). Furthermore, T cell–mediated immunity plays an important role in the protection induced by NS1-based DNA vaccines against ZIKV infection (23). Thus, robust induction of anti-NS1 antibodies associated with activation of T cell responses represent key features of a NS1-based vaccine capable to generate protective immunity to ZIKV.

The protective role of anti-NS1 antibodies has been correlated to the binding activity to surface–associated NS1 in ZIKV-infected cells, which promotes the clearance of the infected cells via ADCC or FcR-mediated complement (FC) cytotoxicity (36–39). Our data indicate that although similar serum anti-NS1 IgG responses were induced with the tested NS1-based DNA vaccines in immunocompetent mice, pgDNS1-ZIKV-vaccined mice generated anti-NS1 antibodies with higher cell binding activity compared to those immunized with pNS1-ZIKV. Since these antibodies are capable to recognize membrane-associated NS1 dimers in ZIKV-infected cells, we expect that, under *in vivo* conditions, this would favor the clearance of infected cells. The presence of higher IgG2c levels in pgDNS1-ZIKV immunized AB6 mice also supports this hypothesis, since IgG2c antibodies are known to strongly bind to ADCC-mediator FcyRIV (67, 68). Thus, the enhanced immunity observed in this group may be related to the role of gD on the modulation of the induced antigen-specific responses. Moreover, cell transfection with pgDNS1-ZIKV increases the *in vitro* expression of NS1 compared to pNS1-ZIKV-transfected cells, a characteristic that may also impact the immunogenicity of the encoded proteins. These findings highlight the adjuvant effects of gD on the immunogenicity of antigens encoded by DNA vaccines.

The relevance of T cells in the control of ZIKV infection has been highlighted in different studies. This type of immune response was recently linked to protection to ZIKV challenge at experimental conditions, but also in the context of protection to secondary heterologous flavivirus infections (58, 69, 70). Moreover, T cell mediated immune responses induced by NS1-vaccination have being described as a key feature in the control of ZIKV infections under preclinical conditions (23, 37). Similar findings have also been reported in clinical studies demonstrating that NS1 is capable to induce robust IFN-γ production by T cells during ZIKV infection (71, 72). Since gD antigen-fusion platform has been extensively explored to increase T cell mediated protective immune responses (40, 41, 46–48), here we tested whether, under a different vaccine context, such approach would lead to enhancement of NS1-specific IFN-γ producing T cells. Using MHC-I restrict peptides of ZIKV NS1 to activate *in vitro* spleen cells of immunocompetent C57BL/6 immunized mice, we observed that only expression of NS1 fused to gD protein promoted significant enhancement of IFN-γ secreting cell responses. Since we used MHC-I restrict peptides to stimulate the cells *in vitro*, these results also suggest activation of CD8^+^ T cell responses in vaccinated mice and open perspectives for the use of the identified MHC-I-restricted peptides in future studies dealing with induction of NS1-specific T cell responses under experimental conditions. Taken together, these findings indicated that gD fusion strategy is a promising alternative to enhance NS1-specific cellular response induced by vaccination.

Our data also corroborate with a recent study reporting the use of DNA vaccines encoding the ZIKV NS1 protein (23). Despite the differences in the tested vaccine regimen, including number of doses (total of three), immunization route (intradermal) and mouse genetic background (BALB/c), only expression of a modified form of NS1 (pVAX-tpaNS1) was able to induce high titers of antibodies capable of recognizing NS1 on infected cells and significant activation of T cell responses. Moreover, the authors reported the presence of immunodominant CD4^+^ and CD8^+^ T cell epitopes at the C-terminal region of NS1 (aa 172 to 352) (23). Similarly, the epitopes restricted to MHC-I predicted and validated in our work are mapped in the same region of the protein. In addition, the genetic fusion with HSV-1 gD protein led to enhanced activation of NS1-specific IFN-γ secreting cells in vaccinated mice. The differences in the CD8^+^ T cell epitopes used in the two studies are reflect the distinct genetic backgrounds of the tested vaccinated mouse strains (BALB/c × C57BL/6). On the other hand, since the validation of the peptides described in our study was performed after infection with ZIKV, these findings may reflect part of repertoire of immunodominant epitopes detected in more natural conditions. Nonetheless, since we did not evaluate the induced immune responses with the full length NS1, other epitopes may also be targeted after immunization with pgDNS1-ZIKV. Similarly, further studies shall address the positive impacts of gD antigen fusion with the induction of NS1-specific CD4^+^ T cell responses.

Wild type (WT) mice, such as C57BL/6, have preserved type I IFN responses that provide defense against flaviviruses infections and, consequently, do not permit efficient virus replication, generation of morbidity and lethality effects related to ZIKV infection (73, 74). To access the protective capacity of the formulations tested here we used a type I IFN receptor–deficient (IFNAR1–/–) mouse strain, which is susceptible to ZIKV infection (57). Adopting the same vaccination protocol, we detected higher serum anti-NS1 IgG titers in mice immunized with pgDNS1-ZIKV when compared to mice immunized with pNS1-ZIKV. After challenge, pgDNS1-ZIKV-vaccinated mice showed reduced morbidity and lethality scores compared to animals immunized with pNS1-ZIKV. In addition, both NS1-based DNA vaccines induced predominant serum IgG2c subclass response and reduced viremia after infection. Taken together, these observations confirm the adjuvant effects of the gD-fusion strategy applied to DNA vaccines, leading to enhanced NS1-specific humoral and cellular immune responses in immunocompetent mice and protective immunity in AB6 mice. The observed NS1-mediated protective profiles are aligned with previous studies dealing with ZIKV NS1 based in immunodeficient mice (35), as well as in immunocompetent BALB/c (23, 60) or CD-1/ICR (37) mice. Moreover, in contrast to the findings described by a study using NS1-based DNA vaccine (23), our formulations was capable to confer protection in IFNAR1^−/−^ mice even after challenge with a high infection dose (10^6^ PFU).

Despite the advantages of DNA vaccines with regard to other vaccine approaches, which includes manufacturing and costs, DNA vaccines usually show reduced immunogenicity when tested at clinical conditions (75). Electroporation represent one of the most effective strategy to enhance the immunogenicity of DNA vaccines both in mice and humans (76). Previous evidences demonstrated that electroporation enhance activation of T-cell mediated protective responses in a murine tumor challenge model (77). In the present study, administration of the DNA vaccines via electroporation contributed to the induction of immune responses with reduction of vaccine doses administered in the animals with regard to other study based on DNA vaccine encoding ZIKV NS1 (23). Altogether, the present evidences further support the relevance of electroporation as a preferred delivery method for administration of DNA vaccines either at experimental or clinical conditions.

Although the adjuvant role of gD is evident in all strategies explored so far, the mechanisms related to the observed adjuvant effects remain not totally elucidated. The gD immunomodulatory effects are mainly based on the binding capacity to specific receptors located on the surface of antigen presenting cells, such as the HVEM receptor in dendritic cells (DC) (48). Binding of gD interferes with interaction of HVEM to its native immunosuppressive ligands leading to final enhancement of B and T cells activation (48). gD binding to HVEM also triggers the activation of NF-kappa B pathway promoting pro-survival signals in T cells (44, 45, 78). Additionally, as previously described, purified chimeric gD-based proteins are capable to activate a DC subset specialized in antigen cross-presentation and leading to enhance generation of activated antigen-specific CD8^+^ T lymphocytes (41). These effects are in line with the results reported here in which generation of a chimeric NS1/gD protein promoted enhanced NS1-specific cellular responses.

Altogether, the findings reported here described for the first time the strategy based on the use of DNA vaccines encoding ZIKV NS1 genetically fused with the HSV-1 gD protein. The adjuvant effects observed with the chimeric NS1/gD, regarding induction of NS1-specific cellular and humoral immune responses, support the use of such approach in further attempts to enhance immunity to ZIKV induced by DNA vaccines and open perspectives for the development of effective anti-ZIKV vaccines as well as other flaviruses.

## Data Availability Statement

The original contributions presented in the study are included in the article/Supplementary Material, further inquiries can be directed to the corresponding author.

## Ethics Statement

The animal study was reviewed and approved by Institutional Animal Care and Use Committee (CEUA) of the University of São Paulo (protocol number 96/2016).

## Author Contributions

LP and LF: conceived and designed the experiments. LP, RA, NS, RA-S, AV-C, SP, MC-A, MR-J, MF, and RC-C: performed the experiments. LP and RA: analyzed the data. MR-J and LM: contributed reagents, materials, and analysis tools. LP: prepared the figures. LP, RA, RA-S, MC-A, and LF: wrote the paper. NS, AV-C, SP, MR-J, and MF: revised the manuscript. All authors read and approved the final version of the manuscript.

## Conflict of Interest

The authors declare that the research was conducted in the absence of any commercial or financial relationships that could be construed as a potential conflict of interest.
