# Supplementary material for: Enhanced Immune Responses and Protective Immunity to Zika Virus Induced by a DNA Vaccine Encoding a Chimeric NS1 Fused With Type 1 Herpes Virus gD Protein
Source: Front Med Technol. 2020 Dec 3;2:604160. doi: 10.3389/fmedt.2020.604160 (PMC8757838; doi:10.3389/fmedt.2020.604160)
Supplement: Supplementary file 1 [file Data_Sheet_1.docx]

Supplementary Material


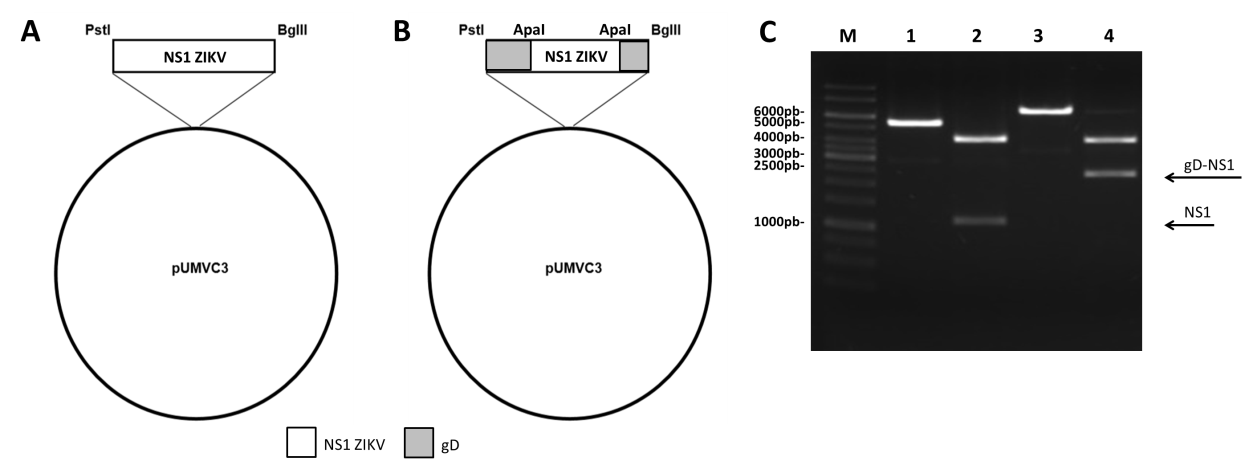


**Supplementary Figure 1.** **Construction of the ZIKV NS1-encoding plasmids**. **(A and B)** Schematic representations of pNS1-ZIKV and pgDNS1-ZIKV and restriction enzyme sites flanking the NS1-ZIKV and gDNS1-ZIKV encoding genes, respectively. **(C)** Restriction analysis with *PstI* and *BglII* enzymes. Electrophoretic mobility of restriction fragments generated after digestion with the restriction enzymes in agarose gel (0.8%). Description: M, Molecular mass marker (SM0314, ThermoFisher); 1, pNS1-ZIKV digested with the *PstI* enzyme; 2, pNS1-ZIKV digested with *PstI* and *BglII*; 3, pgDNS1-ZIKV digested with the *PstI*; 4, pgDNS1-ZIKV digested with *PstI* and *BglII*.


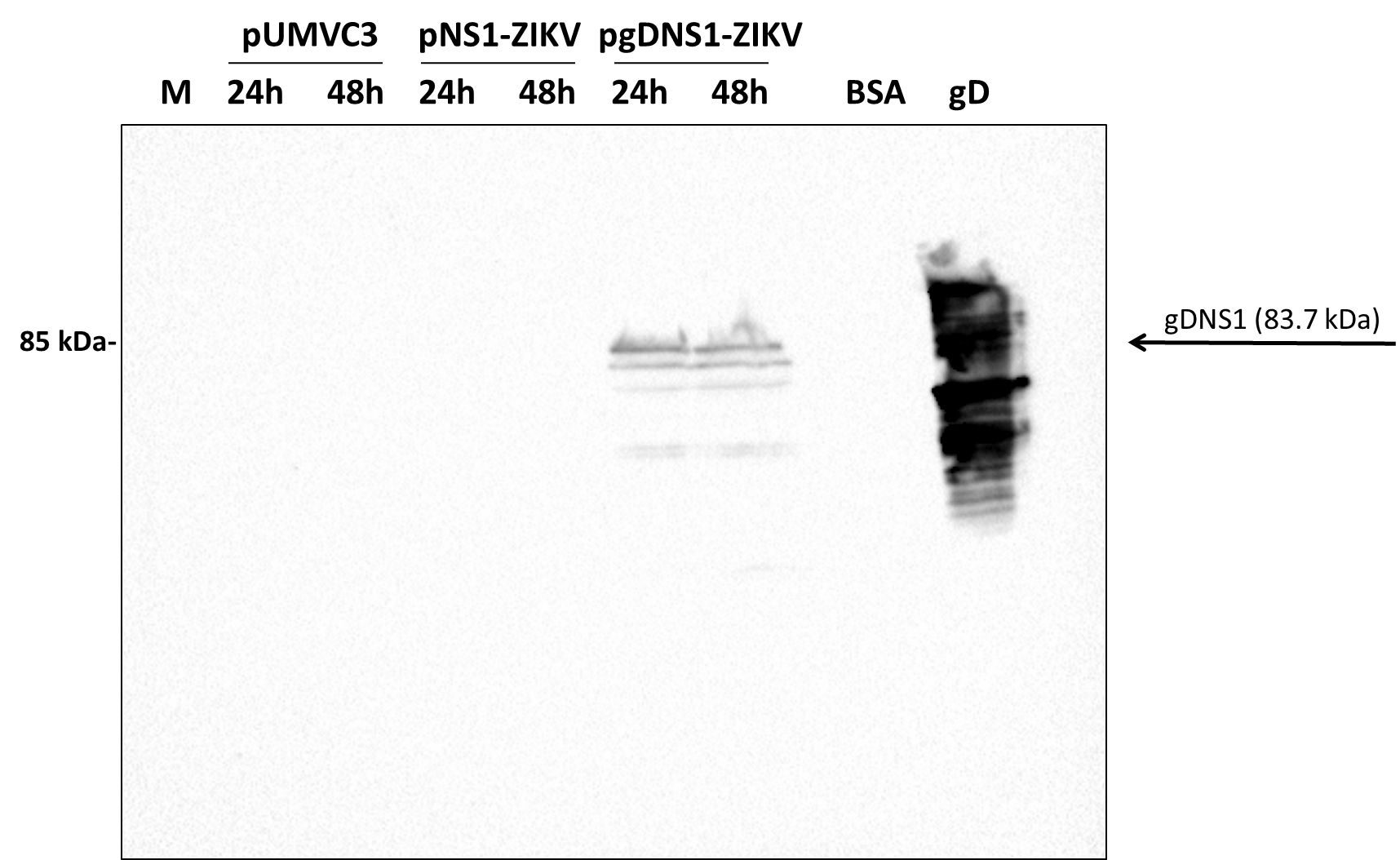


**Supplementary Figure 2.** **Expression of the gD protein in transfected mammalian cells**. HEK293 cells were transfected with plasmids pUMVC3, pNS1-ZIKV or pgDNS1-ZIKV and the expression of gD protein was accessed 24h and 48h after transfection via immunoblot. The cell extracts obtained after lysis were labeled with mAb anti-HSV-I gD followed by HRP-conjugated goat anti-mouse IgG. Description: BSA, bovine serum albumin; gD, recombinant gDE7h protein.


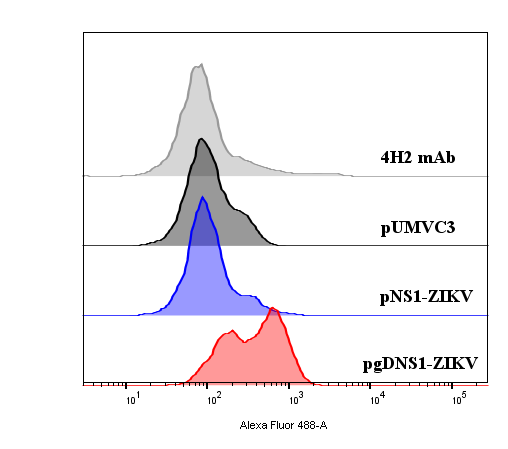


**Supplementary Figure 3.  Binding profile of anti-NS1 serum antibodies to the native ZIKV NS1 expressed on infected cells.** Serum samples collected from vaccinated mice were diluted (1/1000), reacted with ZIKV-infected VERO cells and analyzed by flow cytometry. The 4H2 mAb was used as control.


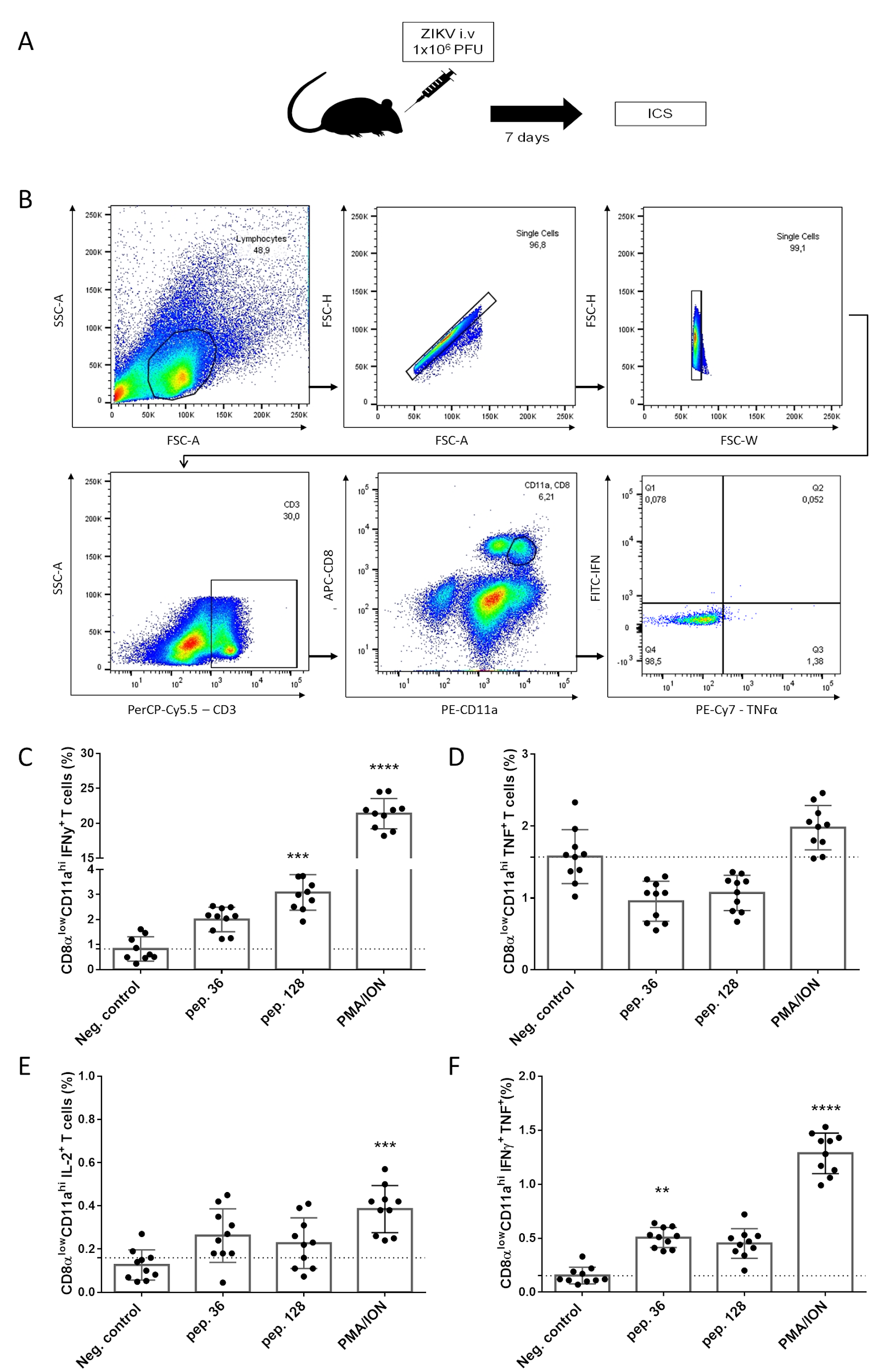


**Supplementary Figure 4.** **Stimulatory activity of the MHC-I restricted peptides based on ZIKV NS1**. C57BL/6 mice were i.v. infected with ZIKV^BR^ and cytokine production was evaluated by intracellular cytokine staining (ICS), seven days after infection (A). Splenocytes isolated from animals were stimulated *in vitro* with each peptide separately. The cells were analyzed by flow cytometry (B) for intracellular accumulation of IFNγ (C), TNFα (D) and IL-2 (E) cytokines. Data are expressed mean ± SD (bars) or individual (symbols) percentage of CD8+ (CD3^+^CD8^low^CD11a^high^) T cells producing the different cytokines. Statistical significance was calculated using One-way ANOVA with Kruskal-Wallis correction. (*p<0.05, **p<0.01, ***p<0.001, ****p<0.0001 compared to the negative control group).

**Supplementary Table 1.** MHC-I restrict peptides derived from the ZIKV NS1 protein.

| Sequence | Length | Position | ID | Strain | Kb | Db |
| --- | --- | --- | --- | --- | --- | --- |
| SHHNTREGYRTQM | 13 | 250-262 | Pep. 36 | ALU33341 | - | X |
| LVRSMVTA | 8 | 343-350 | Pep. 128 | ALU33341 | X | - |
